# Supplementary material for: Protein and DNA Sequence Determinants of Thermophilic Adaptation
Source: PLoS Comput Biol. 2007 Jan 12;3(1):e5. doi: 10.1371/journal.pcbi.0030005 (PMC1769408; doi:10.1371/journal.pcbi.0030005)
Supplement: Supplementary file 1 [file pcbi.0030005.sg001.doc]

**Figure S1**

Dependence of the fraction of KVYWREP amino acids on the optimal growth temperature for the 204 genomes from Table S1.
