# Supplementary material for: Protein and DNA Sequence Determinants of Thermophilic Adaptation
Source: PLoS Comput Biol. 2007 Jan 12;3(1):e5. doi: 10.1371/journal.pcbi.0030005 (PMC1769408; doi:10.1371/journal.pcbi.0030005)
Supplement: Supplementary file 2 [file pcbi.0030005.sg002.doc]

**Figure S2.**

Histogram of the values of the correlation coefficient *R* between OGT and fraction *F* of a set of amino acids for all possible 7-letter combinations (black) and IVYWREL combinations with one substitution (red).
