# Supplementary material for: Protein and DNA Sequence Determinants of Thermophilic Adaptation
Source: PLoS Comput Biol. 2007 Jan 12;3(1):e5. doi: 10.1371/journal.pcbi.0030005 (PMC1769408; doi:10.1371/journal.pcbi.0030005)
Supplement: Supplementary file 3 [file pcbi.0030005.sg003.doc]

**Figure S3**

Probability to find each of the 20 amino acids in the predictor combinations (highest-*R* amino acid combination) calculated from 1000 of 43-organism training sets.
