# Supplementary material for: Protein and DNA Sequence Determinants of Thermophilic Adaptation
Source: PLoS Comput Biol. 2007 Jan 12;3(1):e5. doi: 10.1371/journal.pcbi.0030005 (PMC1769408; doi:10.1371/journal.pcbi.0030005)
Supplement: Supplementary file 5 [file pcbi.0030005.st001.doc]

**Table S1. Cardinal temperatures of prokaryotes (minimum, optimal, and maximum growth temperatures).**

ATCC: American Type Culture Collection, accessed at [http://www.atcc.org](http://www.atcc.org/) in May, 2006

DSMZ: German Collection of Microorganisms and Cell Cultures, accessed at [http://www.dsmz.de](http://www.dsmz.de/) in May, 2006

PGTdb: The Prokaryotic Growth Temperature Database,

S. L. Huang, L. C. Wu, H. K. Laing, K.T. Pan, and J. T. Horng, *Bioinformatics*, Vol. 20, no. 2, pp. 276-278, 2004, accessed at [http://pgtdb.csie.ncu.edu.tw](http://pgtdb.csie.ncu.edu.tw/) in May, 2006

SK, superkingdom (A, archaea; not indicated, bacteria)

|  | **SK** | **Organism** | **OGT, °C** | **T_min_, °C** | **T_max_, °C** | **Source** |
| --- | --- | --- | --- | --- | --- | --- |
| 1 |  | *Acidobacteria bacterium Ellin345* | 25 |  |  | (1) |
| 2 | A | *Aeropyrum pernix* | 95 | 70 | 100 | (2) |
| 3 |  | *Agrobacterium tumefaciens C58 UWash* | 26 |  |  | ATCC |
| 4 |  | *Anabaena variabilis ATCC 29413* | 35 |  |  | (3) |
| 5 |  | *Anaeromyxobacter dehalogenans 2CP-C* | 27 |  |  | ATCC |
| 6 |  | *Aquifex aeolicus* | 85 | 60 | 95 | (4) |
| 7 | A | *Archaeoglobus fulgidus* | 83 | 60 | 95 | (2) |
| 8 |  | *Bacillus anthracis Ames* | 30 |  |  | PGTdb |
| 9 |  | *Bacillus cereus ZK* | 30 |  |  | PGTdb |
| 10 |  | *Bacillus clausii KSM-K16* | 30 |  |  | DSMZ |
| 11 |  | *Bacillus halodurans* | 30 |  |  | DSMZ |
| 12 |  | *Bacillus licheniformis DSM 13* | 37 |  |  | DSMZ |
| 13 |  | *Bacillus subtilis* | 37 |  |  | PGTdb |
| 14 |  | *Bacillus thuringiensis konkukian* | 30 |  |  | PGTdb |
| 15 |  | *Bacteroides fragilis NCTC 9434* | 37 |  |  | DSMZ |
| 16 |  | *Bacteroides thetaiotaomicron VPI-5482* | 37 |  |  | DSMZ |
| 17 |  | *Bartonella henselae Houston-1* | 37 |  |  | PGTdb |
| 18 |  | *Bartonella quintana Toulouse* | 37 |  |  | ATCC |
| 19 |  | *Bdellovibrio bacteriovorus* | 30 |  |  | DSMZ |
| 20 |  | *Bifidobacterium longum* | 37 |  |  | DSMZ |
| 21 |  | *Bordetella bronchiseptica* | 36 |  |  | DSMZ, (5) |
| 22 |  | *Bordetella parapertussis* | 37 |  |  | DSMZ |
| 23 |  | *Bordetella pertussis* | 37 |  |  | DSMZ |
| 24 |  | *Borrelia burgdorferi* | 29 |  |  | (5) |
| 25 |  | *Borrelia garinii PBi* | 35 |  |  | ATCC |
| 26 |  | *Bradyrhizobium japonicum* | 26 |  |  | DSMZ, PGTdb |
| 27 |  | *Brucella melitensis* | 37 |  |  | PGTdb |
| 28 |  | *Burkholderia thailandensis E264* | 30 |  |  | PGTdb |
| 29 |  | *Campylobacter jejuni* | 40 |  |  | DSMZ, PGTdb |
| 30 |  | *Carboxydothermus hydrogenoformans Z-2901* | 67 |  |  | DSMZ |
| 31 |  | *Caulobacter crescentus* | 30 |  |  | DSMZ, PGTdb |
| 32 |  | *Chlamydia muridarum* | 37 |  |  | PGTdb |
| 33 |  | *Chlamydia trachomatis* | 37 |  |  | ATCC |
| 34 |  | *Chlamydophila pneumoniae AR39* | 37 |  |  | ATCC, PGTdb |
| 35 |  | *Chlorobium tepidum TLS* | 48 |  |  | (6) |
| 36 |  | *Chromobacterium violaceum* | 30 |  |  | DSMZ, PGTdb |
| 37 |  | *Chromohalobacter salexigens DSM 3043* | 37 |  |  | (7) |
| 38 |  | *Clostridium acetobutylicum* | 37 |  |  | DSMZ, ATCC |
| 39 |  | *Clostridium perfringens* | 37 |  |  | DSMZ, ATCC |
| 40 |  | *Clostridium tetani E88* | 37 |  |  | DSMZ, PGTdb |
| 41 |  | *Colwellia psychrerythraea 34H* | 8 | -1 | 19 | (8) |
| 42 |  | *Corynebacterium diphtheriae* | 37 |  |  | DSMZ |
| 43 |  | *Corynebacterium efficiens YS-314* | 37 |  |  | DSMZ |
| 44 |  | *Corynebacterium glutamicum ATCC 13032 Bielefeld* | 33 |  |  | DSMZ, ATCC |
| 45 |  | *Corynebacterium jeikeium K411* | 37 |  |  | DSMZ, ATCC |
| 46 |  | *Deinococcus geothermalis DSM 11300* | 47 |  |  | DSMZ |
| 47 |  | *Deinococcus radiodurans* | 30 | 4 | 45 | (9) |
| 48 |  | *Desulfitobacterium hafniense Y51* | 38 |  |  | (10) |
| 49 |  | *Desulfotalea psychrophila LSv54* | 10 | -1.8 |  | (11, 12) |
| 50 |  | *Desulfovibrio desulfuricans G20* | 37 |  |  | DSMZ, ATCC |
| 51 |  | *Desulfovibrio vulgaris Hildenborough* | 30 |  |  | DSMZ, ATCC |
| 52 |  | *Enterococcus faecalis V583* | 37 |  |  | DSMZ |
| 53 |  | *Erwinia carotovora atroseptica SCRI1043* | 26 |  |  | ATCC |
| 54 |  | *Erythrobacter litoralis HTCC2594* | 29 |  |  | DSMZ, ATCC |
| 55 |  | *Escherichia coli K12* | 37 | 10 | 48 | (5, 13) |
| 56 |  | *Francisella tularensis tularensis* | 35 |  |  | (14) |
| 57 |  | *Fusobacterium nucleatum* | 26 |  |  | DSMZ, ATCC |
| 58 |  | *Geobacillus kaustophilus HTA426* | 60 |  | 74 | (15) |
| 59 |  | *Geobacter metallireducens GS-15* | 32 |  |  | (16) |
| 60 |  | *Geobacter sulfurreducens* | 32 |  |  | (17) |
| 61 |  | *Gluconobacter oxydans 621H* | 26 |  |  | DSMZ, ATCC |
| 62 |  | *Haemophilus ducreyi 35000HP* | 32 |  |  | DSMZ, ATCC |
| 63 |  | *Haemophilus influenzae* | 37 |  |  | (5) |
| 64 |  | *Hahella chejuensis KCTC 2396* | 30 | 10 | 45 | (18) |
| 65 |  | *Haloarcula marismortui ATCC 43049* | 37 |  |  | ATCC |
| 66 |  | *Halobacterium sp* | 37 |  |  | ATCC |
| 67 |  | *Helicobacter hepaticus* | 37 |  |  | ATCC |
| 68 |  | *Helicobacter pylori 26695* | 37 |  |  | DSMZ, PGTdb |
| 69 |  | *Idiomarina loihiensis L2TR* | 30 | 4 | 46 | DSMZ, ATCC |
| 70 |  | *Lactobacillus acidophilus NCFM* | 41 |  |  | DSMZ, ATCC |
| 71 |  | *Lactobacillus johnsonii NCC 533* | 37 |  |  | DSMZ, ATCC |
| 72 |  | *Lactobacillus plantarum* | 30 |  |  | DSMZ |
| 73 |  | *Lactobacillus sakei 23K* | 30 |  |  | DSMZ, ATCC |
| 74 |  | *Lactobacillus salivarius UCC118* | 37 |  |  | DSMZ, ATCC |
| 75 |  | *Lactococcus lactis* | 30 |  |  | DSMZ, PGTdb |
| 76 |  | *Legionella pneumophila Philadelphia 1* | 37 |  |  | DSMZ, PGTdb |
| 77 |  | *Leifsonia xyli xyli CTCB0* | 29 |  |  | DSMZ, ATCC |
| 78 |  | *Leptospira interrogans serovar Copenhageni* | 30 |  |  | ATCC |
| 79 |  | *Listeria innocua* | 37 |  |  | DSMZ, ATCC |
| 80 |  | *Listeria monocytogenes* | 37 |  |  | (5) |
| 81 |  | *Magnetospirillum magneticum AMB-1* | 30 |  |  | PGTdb, ATCC |
| 82 |  | *Mesoplasma florum L1* | 30 |  |  | ATCC |
| 83 |  | *Mesorhizobium loti* | 26 |  |  | DSMZ, PGTdb |
| 84 |  | *Methanobacterium thermoautotrophicum* | 65 | 40 | 70 | (19) |
| 85 | A | *Methanococcoides burtonii DSM 6242* | 23 |  |  | DSMZ |
| 86 | A | *Methanococcus jannaschii* | 85 | 50 | 86 | (2) |
| 87 | A | *Methanococcus maripaludis S2* | 35 |  |  | (20) |
| 88 | A | *Methanopyrus kandleri* | 98 | 84 | 110 | (2) |
| 89 | A | *Methanosarcina acetivorans* | 40 | 10 | 50 | (21) |
| 90 | A | *Methanosarcina barkeri fusaro* | 35 |  |  | DSMZ, ATCC |
| 91 | A | *Methanosarcina mazei* | 36 |  |  | DSMZ, ATCC |
| 92 | A | *Methanosphaera stadtmanae* | 37 |  |  | DSMZ |
| 93 | A | *Methanospirillum hungatei JF-1* | 35 |  |  | DSMZ, PGTdb |
| 94 |  | *Methylobacillus flagellatus KT* | 37 |  |  | PGTdb, ATCC |
| 95 |  | *Methylococcus capsulatus Bath* | 37 |  |  | PGTdb, ATCC |
| 96 |  | *Moorella thermoacetica ATCC 39073* | 57 |  |  | DSMZ, ATCC |
| 97 |  | *Mycobacterium avium paratuberculosis* | 39 |  |  | DSMZ, PGTdb |
| 98 |  | *Mycobacterium bovis* | 37 |  |  | DSMZ, ATCC |
| 99 |  | *Mycobacterium leprae* | 37 |  |  | PGTdb |
| 100 |  | *Mycobacterium tuberculosis CDC1551* | 37 |  |  | DSMZ, ATCC |
| 101 |  | *Mycoplasma gallisepticum* | 37 |  |  | ATCC |
| 102 |  | *Mycoplasma genitalium* | 37 |  |  | ATCC, PGTdb |
| 103 |  | *Mycoplasma hyopneumoniae 232* | 37 |  |  | ATCC |
| 104 |  | *Mycoplasma mobile 163K* | 26 |  |  | ATCC |
| 105 |  | *Mycoplasma penetrans* | 37 |  |  | ATCC |
| 106 |  | *Mycoplasma pneumoniae* | 37 |  |  | ATCC |
| 107 |  | *Mycoplasma pulmonis* | 37 |  |  | ATCC |
| 108 |  | *Mycoplasma synoviae 53* | 37 |  |  | ATCC |
| 109 | A | *Nanoarchaeum equitans* | 82 |  |  | (22) |
| 110 | A | *Natronomonas pharaonis* | 41 |  |  | DSMZ, PGTdb |
| 111 |  | *Neisseria gonorrhoeae FA 1090* | 37 |  |  | ATCC, DSMZ |
| 112 |  | *Neisseria meningitidis Z2491* | 37 |  |  | ATCC, DSMZ |
| 113 |  | *Nitrobacter hamburgensis X14* | 28 |  |  | DSMZ |
| 114 |  | *Nitrobacter winogradskyi Nb-255* | 27 |  |  | DSMZ, ATCC |
| 115 |  | *Nitrosococcus oceani ATCC 19707* | 26 |  |  | ATCC |
| 116 |  | *Nitrosomonas europaea* | 26 |  |  | ATCC |
| 117 |  | *Nitrosospira multiformis ATCC 25196* | 26 |  |  | ATCC |
| 118 |  | *Nocardia farcinica IFM10152* | 37 |  |  | DSMZ, ATCC |
| 119 |  | *Novosphingobium aromaticivorans DSM 12444* | 30 |  |  | DSMZ, ATCC |
| 120 |  | *Oceanobacillus iheyensis* | 28 |  |  | DSMZ |
| 121 |  | *Pasteurella multocida* | 37 |  |  | DSMZ, ATCC |
| 122 |  | *Pelobacter carbinolicus* | 30 |  |  | DSMZ |
| 123 |  | *Pelodictyon luteolum DSM 273* | 25 |  |  | DSMZ |
| 124 |  | *Photobacterium profundum SS9* | 15 |  |  | (23) |
| 125 |  | *Photorhabdus luminescens* | 29 |  |  | DSMZ, ATCC |
| 126 | A | *Picrophilus torridus DSM 9790* | 60 |  | 95 | (24) |
| 127 |  | *Porphyromonas gingivalis W83* | 37 |  |  | DSMZ |
| 128 |  | *Propionibacterium acnes KPA171202* | 37 |  |  | DSMZ, ATCC |
| 129 |  | *Pseudoalteromonas haloplanktis TAC125* | 26 |  |  | PGTdb, ATCC |
| 130 |  | *Pseudomonas aeruginosa* | 40 | 8 | 45 | (5) |
| 131 |  | *Pseudomonas fluorescens Pf-5* | 32 | 0 | 35 | (5) |
| 132 |  | *Pseudomonas putida KT2440* | 28 |  |  | (12) |
| 133 |  | *Pseudomonas syringae phaseolicola 1448A* | 26 |  |  | ATCC |
| 134 |  | *Psychrobacter arcticum 273-4* | 22 | -10 | 28 | (25) |
| 135 | A | *Pyrobaculum aerophilum* | 100 |  | 104 | (26) |
| 136 | A | *Pyrococcus abyssi* | 96 | 67 | 102 | (27) |
| 137 | A | *Pyrococcus furiosus* | 100 | 70 | 105 | (2) |
| 138 | A | *Pyrococcus horikoshii* | 98 | 85 | 105 | (28) |
| 139 |  | *Ralstonia metallidurans CH34* | 30 |  |  | ATCC |
| 140 |  | *Ralstonia solanacearum* | 28 |  |  | PGTdb, ATCC |
| 141 |  | *Rhizobium etli CFN 42* | 27 |  |  | DSMZ, ATCC |
| 142 |  | *Rhodobacter sphaeroides 2 4 1* | 30 |  |  | ATCC |
| 143 |  | *Rhodoferax ferrireducens DSM 15236* | 25 |  |  | DSMZ |
| 144 |  | *Rhodopseudomonas palustris BisB18* | 30 |  |  | ATCC |
| 145 |  | *Rhodospirillum rubrum ATCC 11170* | 27 |  |  | DSMZ, PGTdb |
| 146 |  | *Rickettsia conorii* | 35 |  |  | PGTdb |
| 147 |  | *Rickettsia prowazekii* | 35 |  |  | PGTdb |
| 148 |  | *Rickettsia typhi wilmington* | 35 |  |  | PGTdb |
| 149 |  | *Saccharophagus degradans 2-40* | 28 |  |  | DSMZ |
| 150 |  | *Salinibacter ruber DSM 13855* | 37 |  |  | DSMZ |
| 151 |  | *Salmonella enterica Choleraesuis* | 37 |  |  | ATCC |
| 152 |  | *Salmonella typhi* | 37 |  |  | ATCC |
| 153 |  | *Shewanella denitrificans OS217* | 20 |  |  | DSMZ, ATCC |
| 154 |  | *Shewanella oneidensis* | 30 |  |  | ATCC, PGTdb |
| 155 |  | *Shigella boydii Sb227* | 37 |  |  | DSMZ, ATCC |
| 156 |  | *Shigella dysenteriae* | 37 |  |  | ATCC |
| 157 |  | *Shigella flexneri 2a 2457T* | 37 |  |  | ATCC |
| 158 |  | *Shigella flexneri 2a* | 37 |  |  | ATCC |
| 159 |  | *Shigella sonnei Ss046* | 37 |  |  | DSMZ, ATCC |
| 160 |  | *Silicibacter pomeroyi DSS-3* | 29 |  |  | DSMZ, ATCC |
| 161 |  | *Sinorhizobium meliloti* | 26 |  |  | DSMZ, PGTdb |
| 162 |  | *Sodalis glossinidius morsitans* | 28 |  |  | DSMZ |
| 163 |  | *Staphylococcus aureus aureus MRSA252* | 34 |  |  | (5) |
| 164 |  | *Staphylococcus epidermidis ATCC 12228* | 37 |  |  | DSMZ, ATCC |
| 165 |  | *Staphylococcus haemolyticus* | 37 |  |  | DSMZ, ATCC |
| 166 |  | *Staphylococcus saprophyticus* | 37 |  |  | DSMZ, ATCC |
| 167 |  | *Streptococcus agalactiae 2603* | 37 |  |  | ATCC, PGTdb |
| 168 |  | *Streptococcus mutans* | 37 |  |  | ATCC, PGTdb |
| 169 |  | *Streptococcus pneumoniae R6* | 37 | 25 | 42 | (5) |
| 170 |  | *Streptococcus pyogenes M1 GAS* | 37 |  |  | (5) |
| 171 |  | *Streptococcus thermophilus CNRZ1066* | 42 | 20 | 50 | (5) |
| 172 |  | *Streptomyces avermitilis* | 27 |  |  | DSMZ, ATCC |
| 173 |  | *Streptomyces coelicolor* | 27 |  |  | DSMZ, ATCC |
| 174 | A | *Sulfolobus acidocaldarius DSM 639* | 80 | 60 | 85 | (2) |
| 175 | A | *Sulfolobus solfataricus* | 80 |  | 87 | (29, 30) |
| 176 | A | *Sulfolobus tokodaii* | 80 |  |  | (31) |
| 177 |  | *Symbiobacterium thermophilum IAM14863* | 60 | 45 | 65 | (32) |
| 178 |  | *Synechococcus elongatus PCC 7942* | 26 |  |  | ATCC |
| 179 |  | *Thermoanaerobacter tengcongensis* | 75 | 50 | 80 | (33) |
| 180 |  | *Thermobifida fusca YX* | 57 |  |  | (34) |
| 181 | A | *Thermococcus kodakaraensis KOD1* | 95 | 60 | 100 | (35, 36) |
| 182 | A | *Thermoplasma acidophilum* | 59 |  | 65 | (29, 37) |
| 183 | A | *Thermoplasma volcanium* | 60 |  | 65 | (29, 38) |
| 184 |  | *Thermosynechococcus elongatus* | 55 |  |  | (39) |
| 185 |  | *Thermotoga maritima* | 80 | 55 | 90 | (2) |
| 186 |  | *Thermus thermophilus HB27* | 85 |  |  | (40) |
| 187 |  | *Thiobacillus denitrificans ATCC 25259* | 30 |  |  | DSMZ, ATCC |
| 188 |  | *Thiomicrospira crunogena XCL-2* | 25 |  |  | DSMZ, ATCC |
| 189 |  | *Thiomicrospira denitrificans ATCC 33889* | 26 |  |  | ATCC |
| 190 |  | *Treponema denticola ATCC 35405* | 37 |  |  | ATCC |
| 191 |  | *Treponema pallidum* | 37 |  |  | ATCC, PGTdb |
| 192 |  | *Ureaplasma urealyticum* | 37 |  |  | ATCC, PGTdb |
| 193 |  | *Vibrio cholerae* | 37 |  |  | ATCC, PGTdb |
| 194 |  | *Vibrio fischeri ES114* | 28 |  |  | ATCC, PGTdb |
| 195 |  | *Vibrio parahaemolyticus* | 37 |  |  | ATCC |
| 196 |  | *Vibrio vulnificus CMCP6* | 37 |  |  | ATCC |
| 197 |  | *Wolinella succinogenes* | 37 |  |  | DSMZ, ATCC |
| 198 |  | *Xanthomonas campestris* | 26 |  |  | (5) |
| 199 |  | *Xanthomonas citri* | 30 |  |  | PGTdb |
| 200 |  | *Xanthomonas oryzae KACC10331* | 26 | 7 |  | (5) |
| 201 |  | *Xylella fastidiosa* | 26 |  |  | DSMZ, PGTdb |
| 202 |  | *Yersinia pestis KIM* | 28 | 4 | 40 | (41) |
| 203 |  | *Yersinia pseudotuberculosis IP32953* | 37 |  |  | DSMZ, PGTdb |
| 204 |  | *Zymomonas mobilis ZM4* | 27 |  |  | DSMZ, ATCC |

**References for Table S1.**

1.	Janssen, P. H., Yates, P. S., Grinton, B. E., Taylor, P. M. & Sait, M. (2002) *Appl Environ Microbiol* **68,** 2391-6.

2.	Stetter, K. O. (1999) *FEBS Lett* **452,** 22-5.

3.	Kratz, W. A., J. Myers (1955) *American Journal of Botany* **42,** 282-287.

4.	Deckert, G., Warren, P. V., Gaasterland, T., Young, W. G., Lenox, A. L., Graham, D. E., Overbeek, R., Snead, M. A., Keller, M., Aujay, M., Huber, R., Feldman, R. A., Short, J. M., Olsen, G. J. & Swanson, R. V. (1998) *Nature* **392,** 353-8.

5.	(1974) *Bergey's Manual of Determinative Bacteriology* (The Williams & Wilkins Company, Baltimore).

6.	Eisen, J. A., Nelson, K. E., Paulsen, I. T., Heidelberg, J. F., Wu, M., Dodson, R. J., Deboy, R., Gwinn, M. L., Nelson, W. C., Haft, D. H., Hickey, E. K., Peterson, J. D., Durkin, A. S., Kolonay, J. L., Yang, F., Holt, I., Umayam, L. A., Mason, T., Brenner, M., Shea, T. P., Parksey, D., Nierman, W. C., Feldblyum, T. V., Hansen, C. L., Craven, M. B., Radune, D., Vamathevan, J., Khouri, H., White, O., Gruber, T. M., Ketchum, K. A., Venter, J. C., Tettelin, H., Bryant, D. A. & Fraser, C. M. (2002) *Proc Natl Acad Sci U S A* **99,** 9509-14.

7.	Arahal, D. R., Garcia, M. T., Vargas, C., Canovas, D., Nieto, J. J. & Ventosa, A. (2001) *Int J Syst Evol Microbiol* **51,** 1457-62.

8.	Methe, B. A., Nelson, K. E., Deming, J. W., Momen, B., Melamud, E., Zhang, X., Moult, J., Madupu, R., Nelson, W. C., Dodson, R. J., Brinkac, L. M., Daugherty, S. C., Durkin, A. S., DeBoy, R. T., Kolonay, J. F., Sullivan, S. A., Zhou, L., Davidsen, T. M., Wu, M., Huston, A. L., Lewis, M., Weaver, B., Weidman, J. F., Khouri, H., Utterback, T. R., Feldblyum, T. V. & Fraser, C. M. (2005) *Proc Natl Acad Sci U S A* **102,** 10913-8.

9.	Battista, J. R. (1997) *Annu Rev Microbiol* **51,** 203-24.

10.	Bouchard, B., Beaudet, R., Villemur, R., McSween, G., Lepine, F. & Bisaillon, J. G. (1996) *Int J Syst Bacteriol* **46,** 1010-5.

11.	Knoblauch, C., Sahm, K. & Jorgensen, B. B. (1999) *Int J Syst Bacteriol* **49 Pt 4,** 1631-43.

12.	Rabus, R., Ruepp, A., Frickey, T., Rattei, T., Fartmann, B., Stark, M., Bauer, M., Zibat, A., Lombardot, T., Becker, I., Amann, J., Gellner, K., Teeling, H., Leuschner, W. D., Glockner, F. O., Lupas, A. N., Amann, R. & Klenk, H. P. (2004) *Environ Microbiol* **6,** 887-902.

13.	Fong, S. S., Marciniak, J. Y. & Palsson, B. O. (2003) *J Bacteriol* **185,** 6400-8.

14.	Payne, M. P. & Morton, R. J. (1992) *J Vet Diagn Invest* **4,** 264-9.

15.	Takami, H., Takaki, Y., Chee, G. J., Nishi, S., Shimamura, S., Suzuki, H., Matsui, S. & Uchiyama, I. (2004) *Nucleic Acids Res* **32,** 6292-303.

16.	Lovley, D. R., Giovannoni, S. J., White, D. C., Champine, J. E., Phillips, E. J., Gorby, Y. A. & Goodwin, S. (1993) *Arch Microbiol* **159,** 336-44.

17.	Caccavo, F., Jr., Lonergan, D. J., Lovley, D. R., Davis, M., Stolz, J. F. & McInerney, M. J. (1994) *Appl Environ Microbiol* **60,** 3752-9.

18.	Lee, H. K., Chun, J., Moon, E. Y., Ko, S. H., Lee, D. S., Lee, H. S. & Bae, K. S. (2001) *Int J Syst Evol Microbiol* **51,** 661-6.

19.	Smith, D. R., Doucette-Stamm, L. A., Deloughery, C., Lee, H., Dubois, J., Aldredge, T., Bashirzadeh, R., Blakely, D., Cook, R., Gilbert, K., Harrison, D., Hoang, L., Keagle, P., Lumm, W., Pothier, B., Qiu, D., Spadafora, R., Vicaire, R., Wang, Y., Wierzbowski, J., Gibson, R., Jiwani, N., Caruso, A., Bush, D., Reeve, J. N. & et al. (1997) *J Bacteriol* **179,** 7135-55.

20.	Haney, P. J., Badger, J. H., Buldak, G. L., Reich, C. I., Woese, C. R. & Olsen, G. J. (1999) *Proc Natl Acad Sci U S A* **96,** 3578-83.

21.	Sowers, K. R., Baron, S.F., Ferry J.G. (1984) *Applied and Environmental Micribiology* **47,** 971-978.

22.	Huber, H., Hohn, M. J., Stetter, K. O. & Rachel, R. (2003) *Res Microbiol* **154,** 165-71.

23.	Bartlett, D. H. (2002) *Biochim Biophys Acta* **1595,** 367-81.

24.	Futterer, O., Angelov, A., Liesegang, H., Gottschalk, G., Schleper, C., Schepers, B., Dock, C., Antranikian, G. & Liebl, W. (2004) *Proc Natl Acad Sci U S A* **101,** 9091-6.

25.	Bakermans, C., Ayala-Del-Rio, H. L., Ponder, M. A., Vishnivetskaya, T., Gilichinsky, D., Thomashow, M. F. & Tiedje, J. M. (2006) *Int J Syst Evol Microbiol* **56,** 1285-91.

26.	Fitz-Gibbon, S. T., Ladner, H., Kim, U. J., Stetter, K. O., Simon, M. I. & Miller, J. H. (2002) *Proc Natl Acad Sci U S A* **99,** 984-9.

27.	Cohen, G. N., Barbe, V., Flament, D., Galperin, M., Heilig, R., Lecompte, O., Poch, O., Prieur, D., Querellou, J., Ripp, R., Thierry, J. C., Van der Oost, J., Weissenbach, J., Zivanovic, Y. & Forterre, P. (2003) *Mol Microbiol* **47,** 1495-512.

28.	Kawarabayasi, Y., Sawada, M., Horikawa, H., Haikawa, Y., Hino, Y., Yamamoto, S., Sekine, M., Baba, S., Kosugi, H., Hosoyama, A., Nagai, Y., Sakai, M., Ogura, K., Otsuka, R., Nakazawa, H., Takamiya, M., Ohfuku, Y., Funahashi, T., Tanaka, T., Kudoh, Y., Yamazaki, J., Kushida, N., Oguchi, A., Aoki, K. & Kikuchi, H. (1998) *DNA Res* **5,** 55-76.

29.	Schafer, G., Engelhard, M. & Muller, V. (1999) *Microbiol Mol Biol Rev* **63,** 570-620.

30.	She, Q., Singh, R. K., Confalonieri, F., Zivanovic, Y., Allard, G., Awayez, M. J., Chan-Weiher, C. C., Clausen, I. G., Curtis, B. A., De Moors, A., Erauso, G., Fletcher, C., Gordon, P. M., Heikamp-de Jong, I., Jeffries, A. C., Kozera, C. J., Medina, N., Peng, X., Thi-Ngoc, H. P., Redder, P., Schenk, M. E., Theriault, C., Tolstrup, N., Charlebois, R. L., Doolittle, W. F., Duguet, M., Gaasterland, T., Garrett, R. A., Ragan, M. A., Sensen, C. W. & Van der Oost, J. (2001) *Proc Natl Acad Sci U S A* **98,** 7835-40.

31.	Kawarabayasi, Y., Hino, Y., Horikawa, H., Jin-no, K., Takahashi, M., Sekine, M., Baba, S., Ankai, A., Kosugi, H., Hosoyama, A., Fukui, S., Nagai, Y., Nishijima, K., Otsuka, R., Nakazawa, H., Takamiya, M., Kato, Y., Yoshizawa, T., Tanaka, T., Kudoh, Y., Yamazaki, J., Kushida, N., Oguchi, A., Aoki, K., Masuda, S., Yanagii, M., Nishimura, M., Yamagishi, A., Oshima, T. & Kikuchi, H. (2001) *DNA Res* **8,** 123-40.

32.	Ohno, M., Shiratori, H., Park, M. J., Saitoh, Y., Kumon, Y., Yamashita, N., Hirata, A., Nishida, H., Ueda, K. & Beppu, T. (2000) *Int J Syst Evol Microbiol* **50 Pt 5,** 1829-32.

33.	Xue, Y., Xu, Y., Liu, Y., Ma, Y. & Zhou, P. (2001) *Int J Syst Evol Microbiol* **51,** 1335-41.

34.	Bonamore, A., Ilari, A., Giangiacomo, L., Bellelli, A., Morea, V. & Boffi, A. (2005) *Febs J* **272,** 4189-201.

35.	Atomi, H., Fukui, T., Kanai, T., Morikawa, M. & Imanaka, T. (2004) *Archaea* **1,** 263-7.

36.	Fujiwara, S., Takagi, M. & Imanaka, T. (1998) *Biotechnol Annu Rev* **4,** 259-84.

37.	Ruepp, A., Graml, W., Santos-Martinez, M. L., Koretke, K. K., Volker, C., Mewes, H. W., Frishman, D., Stocker, S., Lupas, A. N. & Baumeister, W. (2000) *Nature* **407,** 508-13.

38.	Kawashima, T., Amano, N., Koike, H., Makino, S., Higuchi, S., Kawashima-Ohya, Y., Watanabe, K., Yamazaki, M., Kanehori, K., Kawamoto, T., Nunoshiba, T., Yamamoto, Y., Aramaki, H., Makino, K. & Suzuki, M. (2000) *Proc Natl Acad Sci U S A* **97,** 14257-62.

39.	Nakamura, Y., Kaneko, T., Sato, S., Ikeuchi, M., Katoh, H., Sasamoto, S., Watanabe, A., Iriguchi, M., Kawashima, K., Kimura, T., Kishida, Y., Kiyokawa, C., Kohara, M., Matsumoto, M., Matsuno, A., Nakazaki, N., Shimpo, S., Sugimoto, M., Takeuchi, C., Yamada, M. & Tabata, S. (2002) *DNA Res* **9,** 123-30.

40.	Henne, A., Bruggemann, H., Raasch, C., Wiezer, A., Hartsch, T., Liesegang, H., Johann, A., Lienard, T., Gohl, O., Martinez-Arias, R., Jacobi, C., Starkuviene, V., Schlenczeck, S., Dencker, S., Huber, R., Klenk, H. P., Kramer, W., Merkl, R., Gottschalk, G. & Fritz, H. J. (2004) *Nat Biotechnol* **22,** 547-53.

41.	Bibel, D. J. & Chen, T. H. (1976) *Bacteriol Rev* **40,** 633-51.
