# Supplementary material for: Protein and DNA Sequence Determinants of Thermophilic Adaptation
Source: PLoS Comput Biol. 2007 Jan 12;3(1):e5. doi: 10.1371/journal.pcbi.0030005 (PMC1769408; doi:10.1371/journal.pcbi.0030005)
Supplement: Supplementary file 6 [file pcbi.0030005.st002.doc]

**Table S2**

Combinations of amino acids giving the 10 highest values of the correlation coefficient *R* between OGT and fraction of the amino acids for 86 proteomes.

| Combination | *R* |
| --- | --- |
| ILVWYER | 0.930354 |
| MFILVWYERP | 0.923295 |
| ILVYER | 0.921624 |
| MILVWYER | 0.921488 |
| MFILVYERP | 0.919100 |
| FILVYERP | 0.918807 |
| ILVWYEHR | 0.918141 |
| FILVWYERP | 0.917256 |
| ILVWYGERKP | 0.916480 |
| ILVYEHR | 0.914906 |
