# Supplementary material for: Protein and DNA Sequence Determinants of Thermophilic Adaptation
Source: PLoS Comput Biol. 2007 Jan 12;3(1):e5. doi: 10.1371/journal.pcbi.0030005 (PMC1769408; doi:10.1371/journal.pcbi.0030005)
Supplement: Supplementary file 7 [file pcbi.0030005.st003.doc]

**Table S3**

Organisms used for prediction of transmembrane beta-barrel proteins and their OGT.

| Organism | OGT, °C |
| --- | --- |
| *Aeropyrum pernix* | 95 |
| *Colwellia psychrerythraea 34H* | 8 |
| *Nanoarchaeum equitans* | 82 |
| *Sulfolobus acidocaldarius DSM 639* | 80 |
| *Photobacterium profundum SS9* | 15 |
| *Psychrobacter arcticum 273-4* | 22 |
| *Pyrobaculum aerophilum* | 100 |
| *Thermoanaerobacter tengcongensis* | 75 |
| *Methanococcoides burtonii DSM 6242* | 23 |
| *Desulfotalea psychrophila LSv54* | 10 |
| *Aquifex aeolicus* | 85 |
| *Archaeoglobus fulgidus* | 83 |
| *Bordetella bronchiseptica* | 36 |
| *Borrelia burgdorferi* | 29 |
| *Campylobacter jejuni* | 40 |
| *Chlorobium tepidum TLS* | 48 |
| *Haemophilus ducreyi 35000HP* | 32 |
| *Methanobacterium thermoautotrophicum* | 65 |
| *Methanosarcina acetivorans* | 40 |
| *Methanosarcina mazei* | 36 |
| *Pseudomonas aeruginosa* | 40 |
| *Rickettsia conorii* | 35 |
| *Rickettsia prowazekii* | 35 |
| *Staphylococcus aureus aureus MRSA252* | 34 |
| *Streptomyces coelicolor* | 27 |
| *Sulfolobus solfataricus* | 80 |
| *Thermoplasma acidophilum* | 59 |
| *Thermotoga maritima* | 80 |
| *Yersinia pestis KIM* | 28 |
