# Supplementary material for: Protein and DNA Sequence Determinants of Thermophilic Adaptation
Source: PLoS Comput Biol. 2007 Jan 12;3(1):e5. doi: 10.1371/journal.pcbi.0030005 (PMC1769408; doi:10.1371/journal.pcbi.0030005)
Supplement: Supplementary file 8 [file pcbi.0030005.st004.doc]

**Table S4**

Combinations of nucleotides and correlation coefficients between OGT and fraction of the corresponding nucleotides in the coding DNA of 83 organisms.

| TC | -0.604829 |
| --- | --- |
| C | -0.239541 |
| TGC | -0.225899 |
| GC | -0.147286 |
| AC | -0.122554 |
| AGC | -0.037858 |
| G | -0.014913 |
| ATC | 0.014913 |
| T | 0.037858 |
| TG | 0.122554 |
| AT | 0.147286 |
| A | 0.225899 |
| ATG | 0.239541 |
| AG | 0.604830 |
