# Supplementary material for: Protein and DNA Sequence Determinants of Thermophilic Adaptation
Source: PLoS Comput Biol. 2007 Jan 12;3(1):e5. doi: 10.1371/journal.pcbi.0030005 (PMC1769408; doi:10.1371/journal.pcbi.0030005)
Supplement: Supplementary file 9 [file pcbi.0030005.st005.doc]

**Table S5a**

Correlation coefficients *R* between OGT and fractions of individual amino acids for 83 genomes, ordered by *R*.

| Q | -0.655430 |
| --- | --- |
| H | -0.613252 |
| T | -0.595807 |
| D | -0.452277 |
| C | -0.397283 |
| A | -0.361600 |
| S | -0.321173 |
| N | -0.110072 |
| W | -0.105565 |
| M | -0.088933 |
| G | -0.062129 |
| R | 0.047068 |
| P | 0.057249 |
| L | 0.081959 |
| F | 0.125852 |
| I | 0.248800 |
| K | 0.357022 |
| V | 0.453103 |
| Y | 0.514510 |
| E | 0.677899 |

**Table S5b**

Correlation coefficients *R* between OGT and fractions of two-letter combinations of amino acids for 83 genomes, ordered by *R*.

| VY 0.835 | MY 0.380 | FL 0.163 | CR -0.035 | YQ -0.247 | QP -0.436 |
| --- | --- | --- | --- | --- | --- |
| YE 0.723 | WK 0.377 | IH 0.156 | CL -0.055 | TP -0.254 | AD -0.442 |
| VE 0.708 | SE 0.376 | FR 0.143 | LN -0.056 | AP -0.260 | AT -0.451 |
| WE 0.694 | CY 0.373 | YN 0.136 | CF -0.059 | FD -0.266 | WH -0.461 |
| RK 0.691 | VW 0.369 | YD 0.123 | AE -0.068 | YA -0.273 | WD -0.470 |
| YP 0.667 | FY 0.368 | QE 0.119 | WG -0.074 | GD -0.279 | MH -0.472 |
| FV 0.661 | LV 0.360 | VS 0.116 | CP -0.077 | SP -0.282 | NQ -0.478 |
| EP 0.645 | NE 0.358 | FW 0.113 | HR -0.084 | LA -0.284 | SD -0.484 |
| LE 0.626 | IG 0.353 | IN 0.109 | IQ -0.087 | AG -0.285 | GQ -0.520 |
| VK 0.612 | IY 0.334 | ID 0.096 | MG -0.098 | NH -0.285 | FQ -0.523 |
| ME 0.597 | IL 0.325 | VD 0.092 | NR -0.114 | LT -0.286 | SH -0.529 |
| WY 0.584 | YH 0.321 | YT 0.089 | MN -0.122 | MS -0.291 | AQ -0.529 |
| FE 0.580 | MK 0.321 | LP 0.080 | LH -0.135 | LD -0.294 | WT -0.533 |
| CE 0.566 | CV 0.319 | IT 0.078 | AK -0.137 | ND -0.306 | AS -0.538 |
| EH 0.560 | IK 0.314 | YS 0.078 | DR -0.138 | CM -0.311 | TS -0.547 |
| KP 0.550 | VN 0.313 | IS 0.075 | NP -0.142 | GT -0.318 | MT -0.552 |
| IV 0.547 | FK 0.312 | LR 0.071 | HP -0.146 | QR -0.333 | CD -0.565 |
| EK 0.535 | CK 0.300 | QK 0.056 | WN -0.149 | WA -0.346 | WQ -0.608 |
| IR 0.529 | HK 0.286 | VT 0.055 | FS -0.162 | TN -0.361 | TD -0.609 |
| GK 0.502 | VP 0.282 | MF 0.054 | MW -0.162 | GS -0.367 | AN -0.622 |
| GE 0.500 | FP 0.265 | RP 0.052 | TR -0.164 | WS -0.376 | DH -0.624 |
| IE 0.496 | IW 0.260 | ML 0.050 | VA -0.170 | LQ -0.377 | MQ -0.661 |
| ER 0.495 | DK 0.229 | LW 0.046 | CG -0.174 | FT -0.386 | SQ -0.666 |
| YG 0.492 | FI 0.228 | WR 0.027 | SR -0.185 | MD -0.387 | CT -0.673 |
| LY 0.489 | TK 0.220 | FG 0.026 | CN -0.197 | GN -0.392 | TH -0.675 |
| YR 0.442 | VR 0.216 | MR 0.025 | AR -0.217 | MA -0.396 | CH -0.682 |
| TE 0.419 | MI 0.210 | WP 0.024 | SN -0.221 | CW -0.398 | QH -0.685 |
| IP 0.418 | VH 0.206 | MP 0.020 | FH -0.223 | IA -0.401 | CQ -0.704 |
| MV 0.415 | VG 0.193 | LG 0.005 | DP -0.228 | CA -0.402 | TQ -0.729 |
| YK 0.411 | NK 0.190 | GR 0.003 | GH -0.230 | FA -0.407 | QD -0.782 |
| LK 0.407 | CI 0.185 | GP -0.006 | LS -0.232 | AH -0.411 |  |
| DE 0.387 | SK 0.184 | FN -0.034 | VQ -0.239 | CS -0.428 |  |
